# Design of Pectin-Based Hydrogel Microspheres for Targeted Pulmonary Delivery

**DOI:** 10.3390/gels9090707

**Published:** 2023-09-01

**Authors:** Andy Chai, Keagan Schmidt, Gregory Brewster, Lu Shi Peng Xiong, Benjamin Church, Timothy Wahl, Hamed Sadabadi, Subha Kumpaty, Wujie Zhang

**Affiliations:** 1 Department of Chemistry, Rhodes College, Memphis, TN 38112, USA; chaay-25@rhodes.edu; 2 Chemical and Biomolecular Engineering Program, Department of Physics and Chemistry, Milwaukee School of Engineering, Milwaukee, WI 53202, USA; schmidtk@msoe.edu (K.S.); brewsterg@msoe.edu (G.B.); xiongl@msoe.edu (L.S.P.X.); 3 Advanced Analysis Facility, College of Engineering & Applied Science, University of Wisconsin—Milwaukee, Milwaukee, WI 53211, USA; church@uwm.edu (B.C.); sadabadi@uwm.edu (H.S.); 4 Materials Science & Engineering Department, University of Wisconsin—Milwaukee, Milwaukee, WI 53211, USA; 5 School of Freshwater Sciences, University of Wisconsin—Milwaukee, Milwaukee, WI 53204, USA; wahlt@uwm.edu; 6 Department of Mechanical Engineering, Milwaukee School of Engineering, Milwaukee, WI 53211, USA; kumpaty@msoe.edu

**Keywords:** pectin, gelatin, hydrogel, microspheres, electrospray, drug delivery, pulmonary drug delivery, targeted drug delivery

## Abstract

Pulmonary drug delivery via microspheres has gained growing interest as a noninvasive method for therapy. However, drug delivery through the lungs via inhalation faces great challenges due to the natural defense mechanisms of the respiratory tract, such as the removal or deactivation of drugs. This study aims to develop a natural polymer-based microsphere system with a diameter of around 3 μm for encapsulating pulmonary drugs and facilitating their delivery to the deep lungs. Pectin was chosen as the foundational material due to its biocompatibility and degradability in physiological environments. Electrospray was used to produce the pectin-based hydrogel microspheres, and Design-Expert software was used to optimize the production process for microsphere size and uniformity. The optimized conditions were determined to be as follows: pectin/PEO ratio of 3:1, voltage of 14.4 kV, distance of 18.2 cm, and flow rate of 0.95 mL/h. The stability and responsiveness of the pectin-based hydrogel microspheres can be altered through coatings such as gelatin. Furthermore, the potential of the microspheres for pulmonary drug delivery (i.e., their responsiveness to the deep lung environment) was investigated. Successfully coated microspheres with 0.75% gelatin in 0.3 M mannitol exhibited improved stability while retaining high responsiveness in the simulated lung fluid (Gamble’s solution). A gelatin-coated pectin-based microsphere system was developed, which could potentially be used for targeted drug delivery to reach the deep lungs and rapid release of the drug.

## 1. Introduction

Pulmonary drug delivery is a noninvasive means of drug delivery that is currently being researched as a viable method of treating a multitude of diseases, such as infections. Its unique characteristics allow for rapid delivery, immediate contact with the infection site, and lower doses. Prompt delivery and contact with the infection site are crucial for quickly and efficiently eliminating infections, as they prevent microorganisms from developing resistance to antibiotics [1]. However, administering drugs via the lungs is notably complex due to the evolving defense mechanisms of the respiratory tract, which prevent the entry of inhaled substances into the lungs and eliminate deposited substances [2]. Inhaled drugs can more readily access the upper airways, but the deep lung tissue may encounter challenges such as mucus hypersecretion, thickening, alterations in airway diameter, fibrosis, and inadequate blood circulation [3]. Additionally, there is a possibility for undissolved drug particles to engage with alveolar macrophages or proteolytic enzymes, leading to their inactivation [4]. Optimization of particle formulations is essential for successful delivery to the deep lungs. For this study, hydrogel formulations were used rather than standard microspheres due to their mucoadhesive features, which allow the spheres to penetrate barriers [5]. Additionally, the swelling properties of hydrogels reduce the possibility of macrophage clearance [6]. Hydrogels also exhibit physical properties, such as high water content and low surface tension, that are similar to those of living tissues [7].

The essential factor in overcoming these defenses is the size of the microspheres. Larger particles tend to be deposited in the upper parts of the respiratory tract, so they are unable to reach infections that affect the deep parts of the lung, such as the alveoli. On the other hand, smaller particles are easily exhaled. Currently, attention has been directed towards enhancing aerosol dispersion and producing small particles to achieve precise targeting within the deep regions of the lungs [8]. To achieve targeted pulmonary drug delivery to the deep lungs, the suggested optimal particle size for inhalation is <5 μm, and particles smaller than 3 μm have a 50–60% chance of reaching the alveolar regions [9]. An aerodynamic diameter between 1 and 3 μm is ideal for reaching the respiratory zone in the deep lungs [10].

To produce the microspheres, electrospray technology was used. Electrospray offers several advantages over traditional emulsification methods, allowing for a cost-effective and efficient method to manufacture uniform and stable microspheres as drug carriers [11]. Electrospray employs an electric field to form destabilizing forces within a droplet that is held together by surface tension. Once these destabilizing forces overcome the surface tension, micro-spherical droplets fall and collect onto a conductive sheet [12].

The interstitial fluid within the deep lung environment maintains a slightly alkaline arterial pH, between 7.38 and 7.43 [13]. This alkalinity allows the lungs to host an innate defense against pathogens, as opposed to an acidic pH, which may promote bacterial growth, antibiotic deactivation, and the development of resistance [14]. However, inhaled therapies still encounter challenges, such as enzymatic degradation and rapid lung clearance [15]. Therefore, it is advantageous to develop formulations that can respond effectively to the conditions within the deep lung environment, aiming for targeted pulmonary drug delivery.

Previously, pectin-based hydrogels were regarded as promising candidates in the drug delivery field. However, its use in pulmonary drug delivery has not been explored [16]. For direct pulmonary administration, many therapeutic substances, ranging from antibiotics to antibodies, have been incorporated into hydrogel systems [17,18,19]. For example, hydrogel-based particles with natural polymers, such as Zn^2+^-crosslinked alginate microparticles, for the pulmonary delivery of drugs have been previously investigated [6]. Hydrogels are considered an ideal delivery system due to their high biocompatibility, biodegradability, and low toxicity [7]. This study, for the first time, explored the development of pectin-based hydrogel microspheres with a diameter of 3 μm that could be used to reach the deep parts of the lungs and how their mechanical properties and stability can be improved through coating with polymeric materials, such as gelatin. Microspheres have been widely explored as a potential vehicle for delivering drugs to the lungs through either intravenous administration or inhalation [6]. Size-based targeting of the deep alveoli has been demonstrated using biodegradable poly lactic-co-glycolic acid (PLGA) microspheres with the incorporation of dipalmitoyl phosphatidyl choline (DPPC) [6]. Sub-micron chitosan spheres have also been explored as a carrier used in pulmonary administration for their biodegradability, biocompatibility, low toxicity, and positive charge [20]. Pectin, a structural heteropolysaccharide that forms a significant portion of cell walls in plants, was chosen as the base material due to its biocompatibility and its ability to accommodate the incorporation of drugs or other substances within the polymer [21,22]. Moreover, it is relatively easy and cost-effective to produce pectin-based hydrogel microspheres, such as through electrospray. The primary uses of pectin-based microspheres involve controlled drug delivery, particularly targeted at colon and digestive tract therapies, showcasing anticancer and antibiotic activities [16,23,24]. The basis of ionic pectin-based hydrogel formation can be attributed to the electron-rich hydroxyl and carboxyl groups of pectin, which can coordinate with polyvalent metal ions such as Ca^2+^. The hydroxyl and carboxyl groups of pectin can be used for further modification, such as through the carboxyl-amino group reaction [25]. Low-methoxyl pectin was used because of its solubility and gel-forming characteristics, determined by its degree of esterification of galacturonic acid residues [16]. The abundant presence of anionic carboxylic groups in LM pectin enables the formation of robust polymeric hydrogel networks and facilitates chemical modifications with cationic molecules. Previous studies have shown that pectin-based microspheres, cross-linked using divalent cations such as Ca^2+^, are responsive to pH due to the protonation/deprotonation of the carboxyl groups [26]. Polyethylene oxide (PEO) was used to increase the viscosity of the pectin solution. Previous studies have shown that adding a significant amount of synthetic polymers such as PEO can improve the stability of the jet and facilitate the electrospray/electrospinning of the pectin solution [27]. Pluronic F-127 (a nonionic surfactant), a triblock copolymer of PEO and polypropylene oxide (PPO), was used to reduce the surface tension of the pectin solution. When Pluronic F-127 is present in lower concentrations, it allows for easier formation of smaller droplets during the electrospray process [28]. Ions such as phosphates can also interrupt the Ca-pectin hydrogel network due to the chelation of Ca^2+^ [29,30]. Gelatin, partially hydrolyzed collagen, was chosen as the coating material due to its ability to form polyelectrolyte complexes that stabilize the microsphere structure with pectin, a molecule with many positively charged residues [29]. Gelatin consists of matrix metalloproteinase-degradation sites, which enable gradual degradation under physiological conditions [31]. The positive charge is preferential due to its adherence to epithelial cells via electrostatic forces, avoiding the removal of microspheres by macrophages [32]. Gelatin has previously been used in the formulation of microspheres for targeted drug delivery of chemotherapeutic drugs, functioning as a natural and biodegradable component of the manufacturing process [33]. Additionally, a gelatin matrix was successfully constructed and applied in pulmonary delivery due to its suitability for pulmonary delivery and efficiency in loading drugs [34]. Gelatin’s high biodegradability also makes it an appealing choice, as upon delivery to the site of infection, the microspheres are expected to disintegrate.

**Scheme 1:**
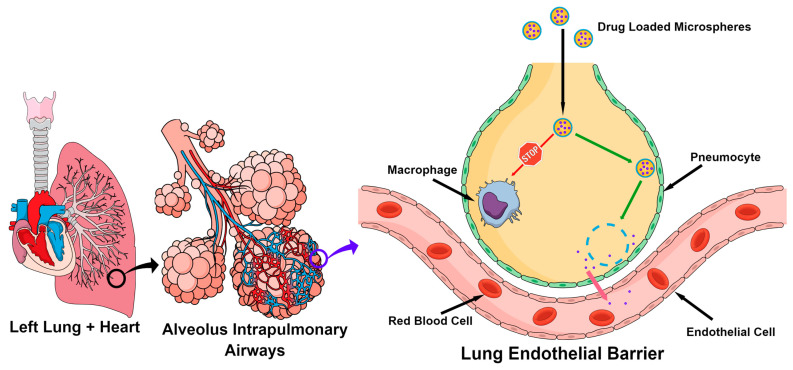
Schematic depicting the mechanism of using gelatin-coated pectin-based hydrogel microspheres for targeted pulmonary drug delivery: upon reaching the alveoli, microspheres retain and dissociate, releasing the drug for absorption before they are cleared, such as by macrophages.

The objective of this study was to develop gelatin-coated pectin-based hydrogel microspheres of ~3 μm, which included optimizing the microsphere production process, formulation, and coating conditions. Upon administration, the size and surface charge promote the retention of microspheres in the deep lungs. Due to the responsiveness of the microspheres to the interstitial lung environment (e.g., slightly alkaline pH and phosphate ions), microspheres disintegrate and release the drugs for absorption before they are cleared by macrophages (Scheme 1).

## 2. Results and Discussion

### 2.1. Hydrogel Microsphere Production Process Optimization

**Figure 1:**
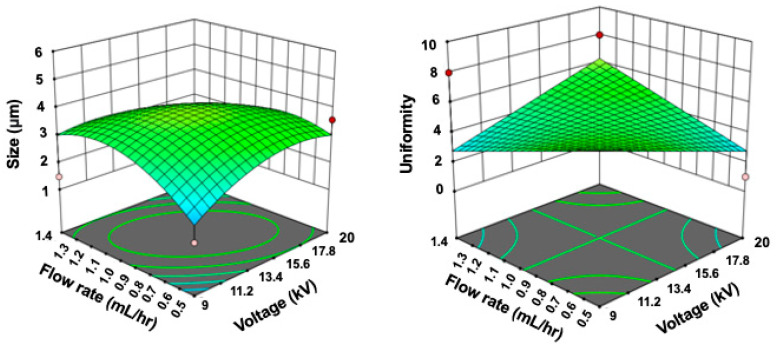
Surface response curves showing the impacts of the most significant interaction on microsphere size (**left**) and uniformity (**right**). Dark red dots indicate design points above the predicted value, while pink dots denote design points below the predicted value.

**Figure 2:**
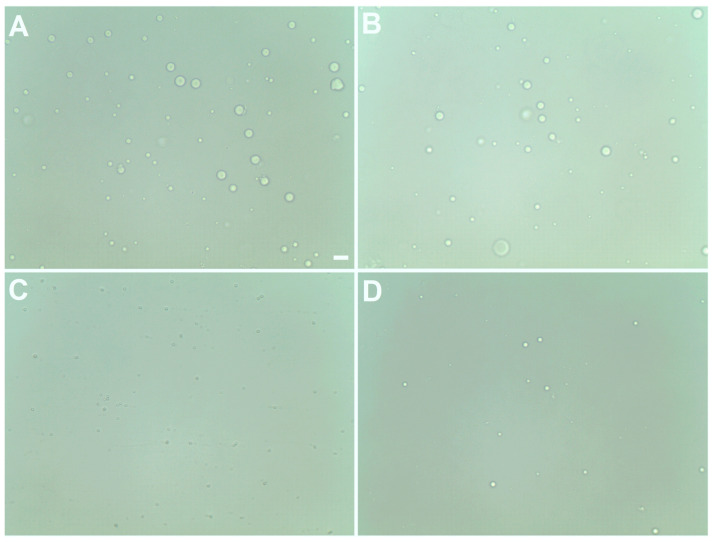
Representative images of microspheres during different trials in the optimization process. (**A**) Undesired size and non-uniform distribution (pectin/PEO ratio of 70:30, voltage of 14.5 kV, 12.5 cm distance, and 1.4 mL/h flow rate). (**B**) Desired size but non-uniform distribution (pectin/PEO ratio of 65:35, voltage of 14.4 kV, 18.2 cm distance, and 0.95 mL/h flow rate). (**C**) Undesired size but uniform distribution (pectin/PEO ratio of 80:20, voltage of 14.5 kV, 20.0 cm distance, and 1.4 mL/h flow rate). (**D**) Desired size and uniform distribution (pectin/PEO ratio of 90:10, voltage of 14.5 kV, 12.5 cm distance, and 1.4 mL/h flow rate). The scale bar represents 20 μm.

It is challenging to produce pectin-based hydrogel microspheres < 200 μm using electrospray. Preliminary studies indicated that adding PEO and Pluronic F-127 can improve the electrospray process for generating microspheres < 10 μm. To optimize the process for producing small pectin-based hydrogel microspheres, a design of experiment (DOE) software, which applies statistical methods to efficiently optimize processes, Stat-Ease Design-Expert^®^ software wasutilized, and the Box–Behnken model was used. For this study, the total experimental runs were reduced from 81 to 25. During the optimization of the microsphere production process, two key responses were considered: size and uniformity (Figure 1). Figure 2 shows hydrogel microspheres produced under different parameters. The most significant factor affecting the size of the microspheres was the distance (*p* < 0.0001). As the distance increased, there was a consistent trend of larger microsphere diameters. This can be attributed to the electric field decreasing as distance increases, yielding larger microspheres. The pectin/PEO ratio also has a critical impact on the size of the microspheres (*p* = 0.0102). As the percentage of pectin increases, the solution becomes difficult to spray properly due to its low viscosity, resulting in a decreased yield of microspheres and a smaller size than desired. On the other hand, an increase in PEO percentage leads to an increase in viscosity, thereby improving the solution’s electrosprayability. Another factor that affected the size of the microspheres was the flow rate (*p* = 0.03331). As the flow rate increases, the size generally increases due to a larger volume of liquid ejected from the needle tip, which leads to larger droplets. If the flow rate is too low, the spray flow becomes discontinuous, as seen in previous studies [35].

The primary factor affecting uniformity was distance (*p* < 0.0001). As the distance increased, the uniformity rating generally increased. The collection distance influences the strength of the electric field, yielding spheres of varying uniformity. The pectin/PEO ratio also has a significant impact on the uniformity of the microspheres (*p* = 0.0388). As the percentage of pectin increases from the optimal value, the uniformity of the microspheres decreases as the spheres are unable to be sprayed properly due to the low viscosity. Additionally, a trend can be seen where an increase in voltage leads to a decrease in uniformity (Figure 1). An increase in spray voltage leads to a rise in the repulsive force between droplets due to ion movement towards the droplet surface, leading to the instability of the jet at high voltages [35]. As shown in Figure 1, to achieve the best uniformity, a lower flow rate and voltage should be chosen.

The data were represented using quadratic models, as shown in Equation (1), where Y is the value of the response variable, β_0_ is the intercept coefficient, the first β_i_ items represent the linear coefficients, the second β_i_ items represent the quadratic coefficients, and β_ij_ items represent the coefficients of the interaction terms.

(1) $$Y=\beta_{0}+\overset{k}{\underset{i=1}{\sum }}\left(\beta_{i}X_{i}\right)+\overset{k}{\underset{i=1}{\sum }}\left(\beta_{i}X_{i}^{2}\right)+\overset{k-1}{\underset{i=1}{\sum }}\overset{k}{\underset{j>i}{\sum }}(\beta_{ij}X_{i}X_{j})$$

During the optimization of the production process, the software returned the following Equations (2) and (3) based on the input:

(2) $$Y_{1}=3.92-0.6923A-0.6302BD-0.6587B^{2}-2.08C^{2}-0.8762D^{2}$$

(3) $$Y_{2}=4.54+1.75BD-1.75A^{2}+4.38C^{2}$$

where Y1 and Y2 are size and uniformity (morphology), respectively; and A, B, C, and D are independent variables, as follows: pectin/PEO ratio, voltage (kV), distance (cm), and flow rate (mL/h), respectively. The optimized conditions were found to be as follows: pectin/PEO ratio of 3:1, voltage of 14.4 kV, 18.2 cm distance, and 0.95 mL/h flow rate. Both equations (models) are significant, with F-values of 8.11 and 16.46, respectively. The corresponding *p*-values are 0.0003 and <0.0001. To interpret the data, taking the first model (size) as an example, there is only a 0.03% chance that an F-value this large (significant) would occur due to noise. Additionally, the R^2^, adjusted R^2^, predicted R^2^, and adequate precision values for the first model are 0.6809, 0.5970, 0.3728, and 7.2703, respectively. The corresponding values for the second model are 0.7016, 0.6590, 0.4123, and 8.0738.

### 2.2. Effects of Gelatin Concentration and Solvent on Hydrogel Microsphere Coatings

**Figure 3:**
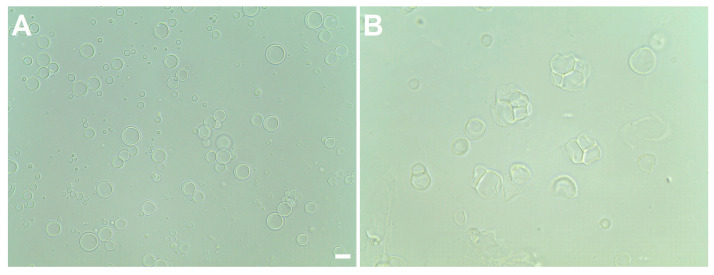
Optical microscopy images of microspheres coated with 0.2% (**A**) and 1.5% (**B**) gelatin in DI water. Microspheres are much larger after coating (**A**) and swollen/collapsed (**B**). The scale bar represents 20 μm.

Type A gelatin was selected due to its higher isoelectric point (pI) compared to type B. Molecules carry an overall positive charge in pH environments lower than their pI. Various gelatin concentrations (0.2–1.5% in DI water) were utilized for optimizing the coating process of microspheres. Unfortunately, significant swelling and/or collapse of the microspheres appeared during the coating process, regardless of the gelatin concentration (Figure 3). This may be due to the osmolarity imbalance [36].

**Figure 4:**
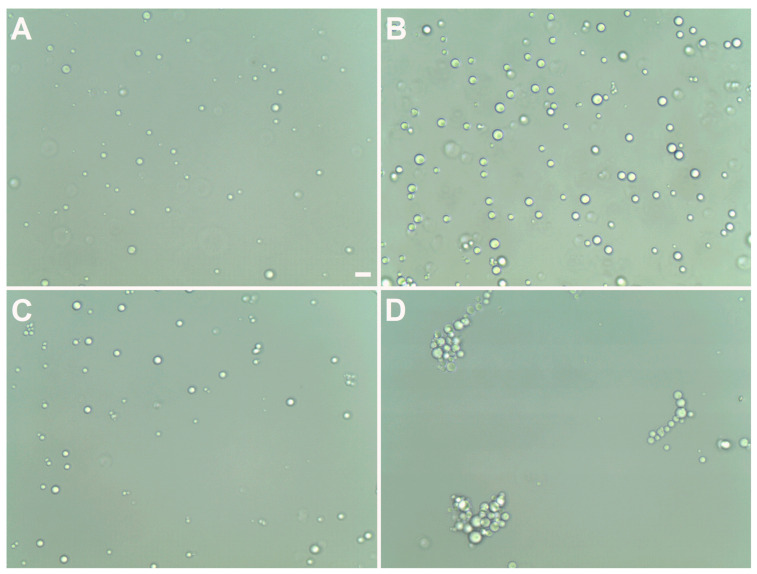
Optical microscopy images of microspheres coated with 0% (**A**), 0.25% (**B**), 0.75% (**C**), and 1.25% (**D**) gelatin in mannitol. The scale bar represents 100 μm. Microspheres significantly increase in size after coating (**B**) and clump together (**D**). The scale bar represents 20 μm.

**Figure 5:**
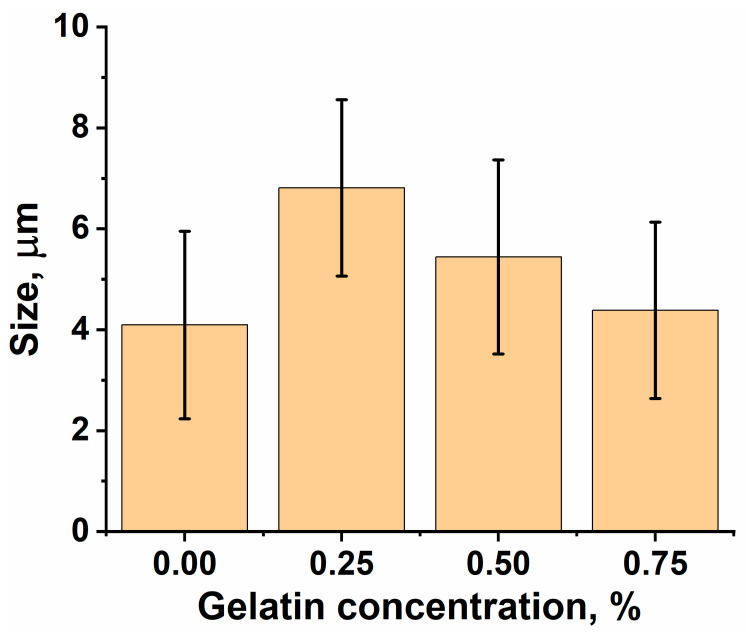
Sizes of hydrogel microspheres coated in different concentrations of gelatin solutions. The data represent the average ± standard deviation. The sizes of microspheres coated with gelatin of higher concentrations are not shown due to clumping.

**Figure 6:**
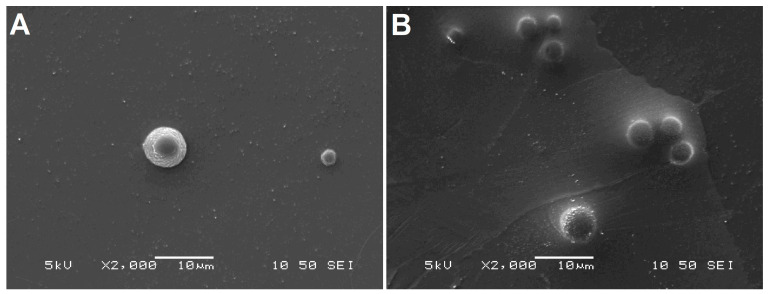
SEM images of PMs (**A**) and PGMs (**B**). The scale bars represent 10 μm.

Mannitol, a sugar alcohol, has been used as an osmotic diuretic to produce an osmotic effect in animals [37]. To overcome this challenge, mannitol was used to help balance osmolarity. Instead of DI water, gelatin was dissolved in 0.3 M mannitol. A previous study successfully used 0.3 M mannitol to help coat Ca-alginate microspheres using chitosan or poly- _L_-lysine (PLL), preventing collapse [36]. The study also revealed that the coating process was notably influenced by the concentration and molecular weight of chitosan. As expected, this study found that the gelatin concentration played a pivotal role in the coating process. As shown in Figure 4 and Figure 5, the microspheres tend to swell if the gelatin concentration is less than 0.75%. At the same time, if the concentration is higher than 0.75%, clumping of the microspheres occurs. The sizes of the microspheres with a gelatin concentration higher than 0.75% are not determined/shown in Figure 5 due to clumping. This can be explained by the occurrence of localized gelation, where crosslinking may take place among the gelatin molecules residing on the surfaces of adjacent microspheres [29]. The 0.75% concentration was determined to be optimal and used for further studies (Figure 6). Overall, the coating process is very different from conventional microspheres, which are usually larger than 200 μm. The result was consistent with a previous studyon coating small (~100 μm) alginate-based microspheres—the incorporation of mannitol solution was critical for maintaining microspheres’ size and morphology [36]. Additionally, the concentration of the coating molecules should be optimized.

### 2.3. Morphology, Surface Properties, and Chemistry of Microspheres

**Figure 7:**
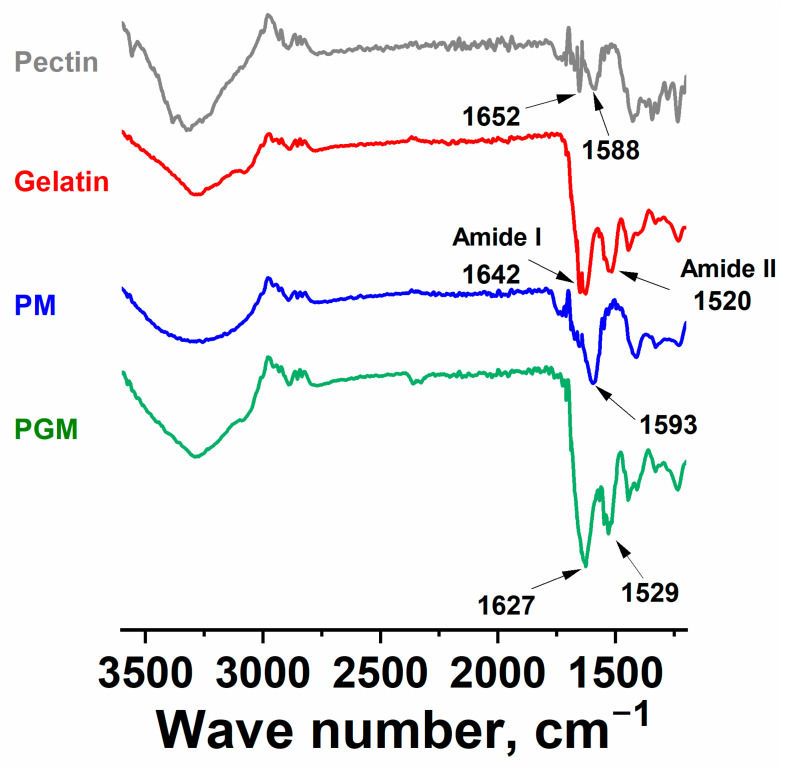
ATR-FTIR spectra (transmission mode) of pectin, gelatin, and hydrogel microspheres.

SEM images of hydrogel microspheres are shown in Figure 6. Uncoated microspheres (PMs) show calcium deposits on the surface and tend to aggregate (Figure S1). Coated microspheres (PGMs) maintain their integrity and have a smoother surface. When it comes to surface properties, as expected, PGMs show a positive surface charge (1.48 ± 0.27  mV) while PMs show a negative value (−2.52 ± 1.11  mV). Again, a positive charge is desired for microsphere retention in the deep lungs. Therefore, positively charged PGMs are more desirable than negatively charged PMs for pulmonary drug delivery. ATR-FTIR was used to study the chemistry of the hydrogel microspheres, and the spectra are shown in Figure 7. Pectin displays peaks at 1652 cm^−1^ (COOH) and 1588 cm^−1^ (COO^−^). Amide I and II bands of gelatin appear at 1642 cm^−1^ and 1520 cm^−1^, respectively. Comparing the spectra of PMs and pectin, the COO^−^ peak is slightly shifted to 1593 cm^−1^, and the peak around 3250 cm^−1^ becomes broader [30,38]. Otherwise, the PM spectrum shows characteristic peaks of the pectin spectrum. The PGM spectrum shows peaks characteristic of both pectin and gelatin. The shift and merging of the peaks of the ionized carboxylic acid of pectin and Amide II of gelatin suggest an interaction between pectin and gelatin. Overall, the FTIR shows the successful gelatin coating of the pectin-based microspheres.

### 2.4. Responsiveness of Hydrogel Microspheres

**Figure 8:**
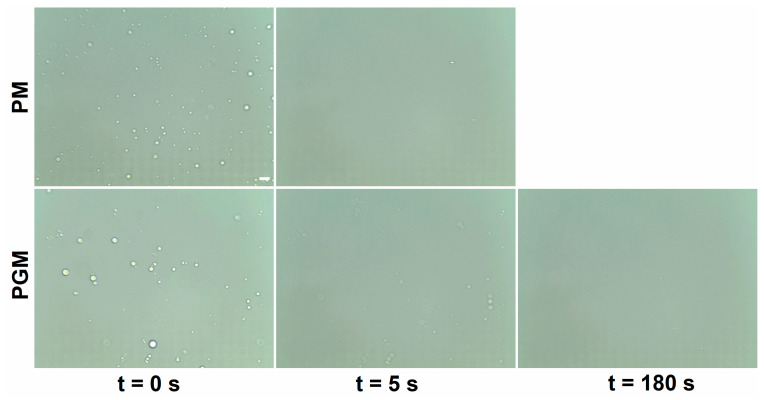
Responsiveness of PMs and PGMs in Gamble’s solution: images of microspheres at different time intervals. The scale bar represents 20 μm.

Gamble’s solution was used to simulate the fluid in the deep lungs. As shown in Figure 8 and Movie S1, PMs dissociate within 5 s when immersed in Gamble’s solution. Gamble’s solution was chosen as the simulated lung fluid because it most closely resembles the composition and salts of the interstitial fluid in the deep lungs [39]. PGMs show higher stability while retaining their responsiveness in Gamble’s solution. Upon the addition of Gamble’s solution, the PGMs swelled in the initial stages. The size changes of the microspheres (PGMs) with time are shown in Figure S2. It took an average of 176.7 (±5.8) seconds for the PGMs to completely dissociate. The results were from five runs of various batches of microspheres.

## 3. Conclusions

Gelatin-coated hydrogel microspheres of ~3 μm exhibit great promise in targeted pulmonary drug delivery applications. During the production of pectin-based hydrogel microspheres (PMs) for coating, it was observed that distance, pectin/PEO ratio, and flow rate significantly impacted microsphere diameter, while microsphere uniformity was most significantly impacted by distance and pectin/PEO ratio. The optimized conditions were determined to be as follows: pectin/PEO ratio of 3:1, voltage of 14.4 kV, 18.2 cm distance, and 0.95 mL/h flow rate. Mannitol is critical to maintaining microsphere size/integrity during gelatin coating. A solution of 0.75% gelatin in 0.3 M mannitol was determined to be optimal for coating the pectin-based hydrogel microspheres. Successful coating of the microspheres in gelatin was confirmed by surface charge (ζ—potential), FTIR, and SEM analysis. More importantly, gelatin-coated microspheres exhibited improved stability but still retained high responsiveness in the simulated lung fluid (Gamble’s solution). Overall, the gelatin-coated pectin-based microspheres (PGMs) show great potential as drug carriers for targeted pulmonary delivery. Future studies will include further characterization of the PGM, such as aerodynamics, biocompatibility, drug loading capacity, and drug release profiles.

## 4. Materials and Methods

### 4.1. Materials

Low-methoxyl pectin was purchased from WillPowder (Miami Beach, FL, USA). Porcine skin gelatin (Type A; G1890), poly(ethylene oxide) (PEO; 182,028), and Pluronic^®^ F-127 (P2443) were acquired from Sigma-Aldrich (St. Louis, MO, USA). All materials were used as received.

### 4.2. Pectin-Based Hydrogel Microsphere Production

To prepare the pectin and PEO solutions, 2 g of each chemical was dissolved in 50 mL of DI water by stirring overnight. After mixing the PEO and pectin solutions at a specific ratio, Pluronic F-127, equivalent to 1% (*w/v*) of the final mass, was then slowly added to the pectin-PEO mixture until fully dissolved. Pectin-based hydrogel microspheres (PMs) were generated using an electrospinning/electrospray setup (Linari Engineering, Valpania, Italy). First, a 3 mL syringe was filled with 2.5 mL of a solution containing various ratios of pectin and PEO, and 1% (*w/v*) Pluronic F-127. The positive electrode was attached to the blunt 30-gauge syringe tip, while the negative electrode was connected to the aluminum foil collecting sheet. After electrospray of the 2.5 mL solution, the microspheres were collected by flushing the aluminum foil sheet using 10 mL of 0.15 M CaCl_2_. Lastly, the microspheres were collected using centrifugation for immediate use or storage at 4 °C. Microspheres were observed under an optical inverted microscope with a 40× objective (EVOS XL; Thermo Fisher Scientific, Waltham, MA, USA). The images were analyzed, including size measurements, using NIH ImageJ software (v1.53t; Bethesda, MA, USA). At least 30 microspheres per sample were analyzed.

### 4.3. Optimization of the Hydrogel Microsphere Production Process

Preliminary studies show that pectin/PEO ratio (70% pectin: 30% PEO, 80% pectin: 20% PEO, 90% pectin: 10% PEO), voltage (9 to 20 kV), flow rate (0.5 to 1.4 mL/h), and distance between the needle tip and the aluminum foil sheet (5 to 20 cm) were determinant factors with working ranges. Design-Expert^®^ software (Version 22.0.3; Stat-Ease Inc., Minneapolis, MN, USA) was used to assist in optimizing the production process based on the Box–Behnken design (BBD) model. A total of 25 trials were performed based on the experimental design of Design-Expert^®^. The two responses used for optimization were size and uniformity. The sizes of the microspheres from the trials were measured using NIH ImageJ software. Uniformity, based on the size deviation from the average, was assessed on a scale of 1–10. The target size was 3 μm (limit range of 2 to 6 μm), and the maximum uniformity rating was 10. The uniformity rating was based on the ratio of the standard deviation to the average diameter of each run. The increments used were 0.05, with 1 being 0.6 and 10 being 0.1. High F-values and low *p*-values indicate that the model is significant. *p*-values less than 0.05 indicate that model terms are significant.

### 4.4. Gelatin Coating of Microspheres

Gelatin was dissolved in a water bath set at 50 °C. Hydrogel microspheres (PMs) were incubated in 0.2–1.5% (*w/v*) gelatin solutions for 15 min and then rinsed twice in DI water [40,41]. Gelatin-coated microspheres (PGMs) were then evaluated under an inverted microscope for optimal gelatin concentration.

### 4.5. Characterization of Microspheres

The zeta potentials of both PMs and PGMs (suspended in DI water) were measured using a Zetasizer (Nano ZS; Malvern Instruments, Westborough, MA, USA). Scanning electron microscopy (SEM) was performed with a JSM-6460 LV Scanning Electron Microscope (JEOL; Tokyo, Japan). To prepare the samples for SEM imaging, microspheres were placed on conductive carbon tape from a liquid suspension and allowed to dry at room temperature. Samples were then sputter-coated with a thin (~2 nm) layer of Au-Pd. SEM imaging was performed with an accelerating voltage of 5 kV. Dried microspheres were analyzed using attenuated total reflection Fourier transform infrared (ATR-FTIR; MIRacle 10, IR-Tracer 100; Shimadzu, Kyoto, Japan) spectroscopy. Transmission mode was used, and the spectra were graphed and analyzed using Origin Pro.

### 4.6. Responsiveness of Hydrogel Microspheres in Simulated Lung Fluid

Gamble’s solution (pH of 7.3–7.4) was used as the simulated lung fluid, which contains 0.116 M NaCl, 0.010 M NH_4_Cl, 0.027 M NaHCO_3_, 0.005 M Glycine, 0.0002 M Trisodium Citrate, 0.0002 M CaCl_2_, 0.001 M _L_-Cysteine, 0.0005 M H_2_SO_4_, and 0.0012 M NaH_2_PO_4_ [42]. Hydrogel microspheres were suspended in Gamble’s solution and evaluated for responsiveness under an inverted microscope.

## Data Availability

The data that support the findings of this study are available from the corresponding author upon reasonable request.

## Supplementary Materials

The following supporting information can be downloaded at: https://www.mdpi.com/article/10.3390/gels9090707/s1, Figure S1: SEM images of aggregated uncoated microspheres (PMs); Movie S1: Responsive of PGMs in Gamble’s solution; Figure S2: Size changes of PGMs in Gamble’s solution.
