# Supplementary material for: Design of Pectin-Based Hydrogel Microspheres for Targeted Pulmonary Delivery
Source: Gels. 2023 Sep 1;9(9):707. doi: 10.3390/gels9090707 (PMC10529711; doi:10.3390/gels9090707)
Supplement: Supplementary file 1 [file gels-09-00707-s001.zip › Supplementary New/Supplementary Material Zhang.pdf]

Supplementary Materials

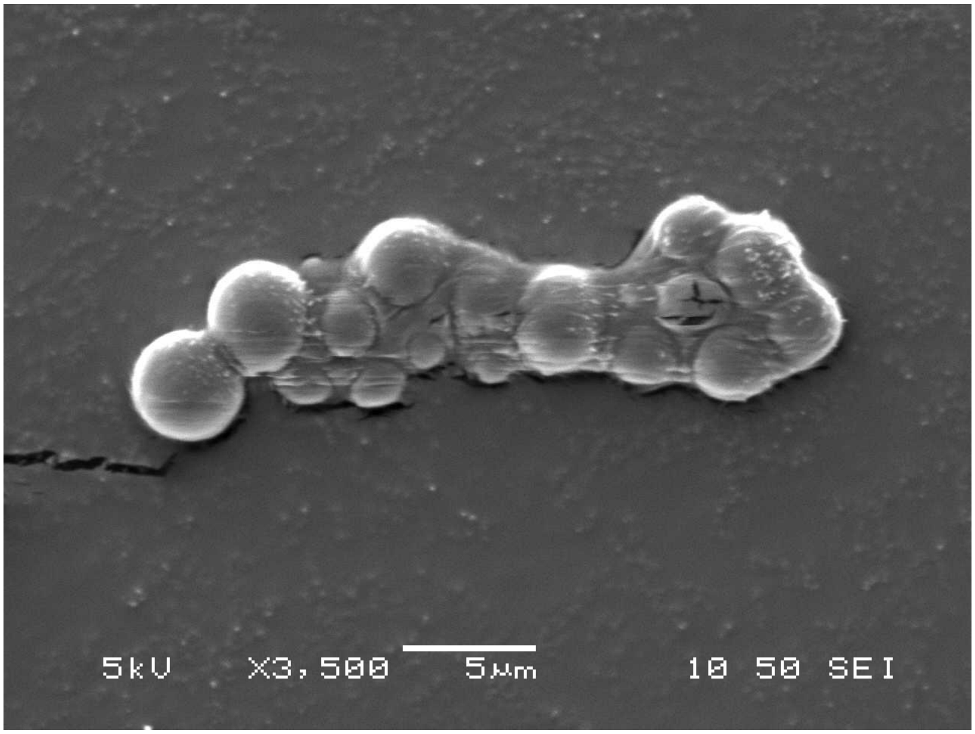

Figure S1: SEM image of aggregated uncoated microspheres (PM). The scale bar represents 5 μm.

Movie S1: Responsiveness of PGM in Gamble's solution.

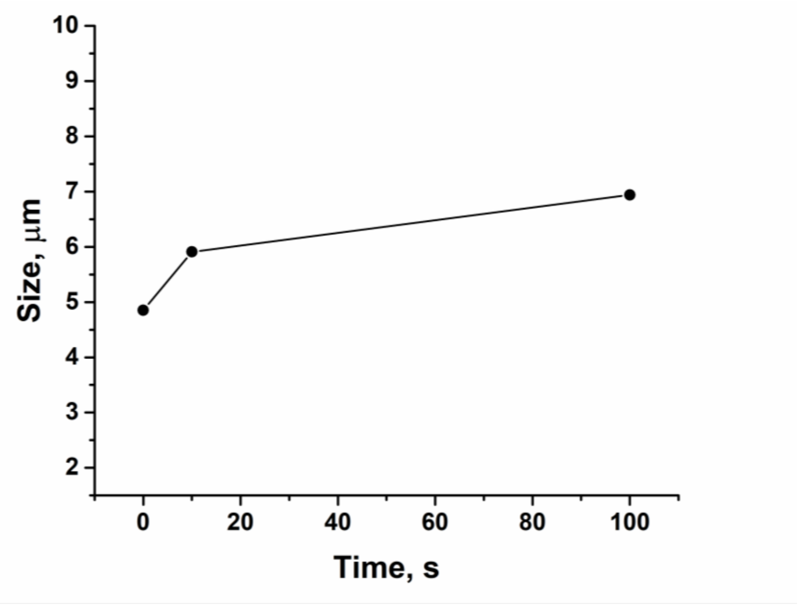

Figure S2: Size changes of PGM in Gamble's solution.
